# Supplementary material for: Irreversible electroporation reverses resistance to immune checkpoint blockade in pancreatic cancer
Source: Nat Commun. 2019 Feb 22;10:899. doi: 10.1038/s41467-019-08782-1 (PMC6385305; doi:10.1038/s41467-019-08782-1)
Supplement: Supplementary file 1 — Supplementary Information [file 41467_2019_8782_MOESM1_ESM.pdf]

Supplementary Information

Irreversible Electroporation Reverses Resistant to Immune Checkpoint Blockade in Pancreatic Cancer

Zhao et al. 2019

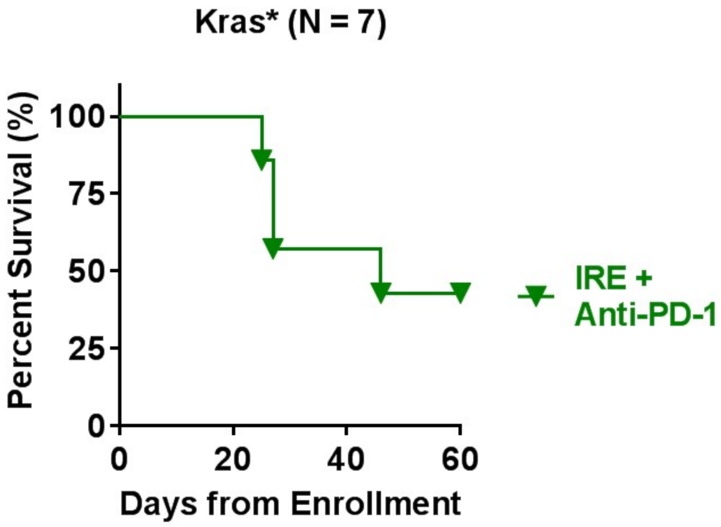

Supplementary Figure 1. Second survival study of KRAS*-bearing mice after treatment with IRE and anti-PD-1. Seven mice were enrolled. Source data are provided as a Source Data file.

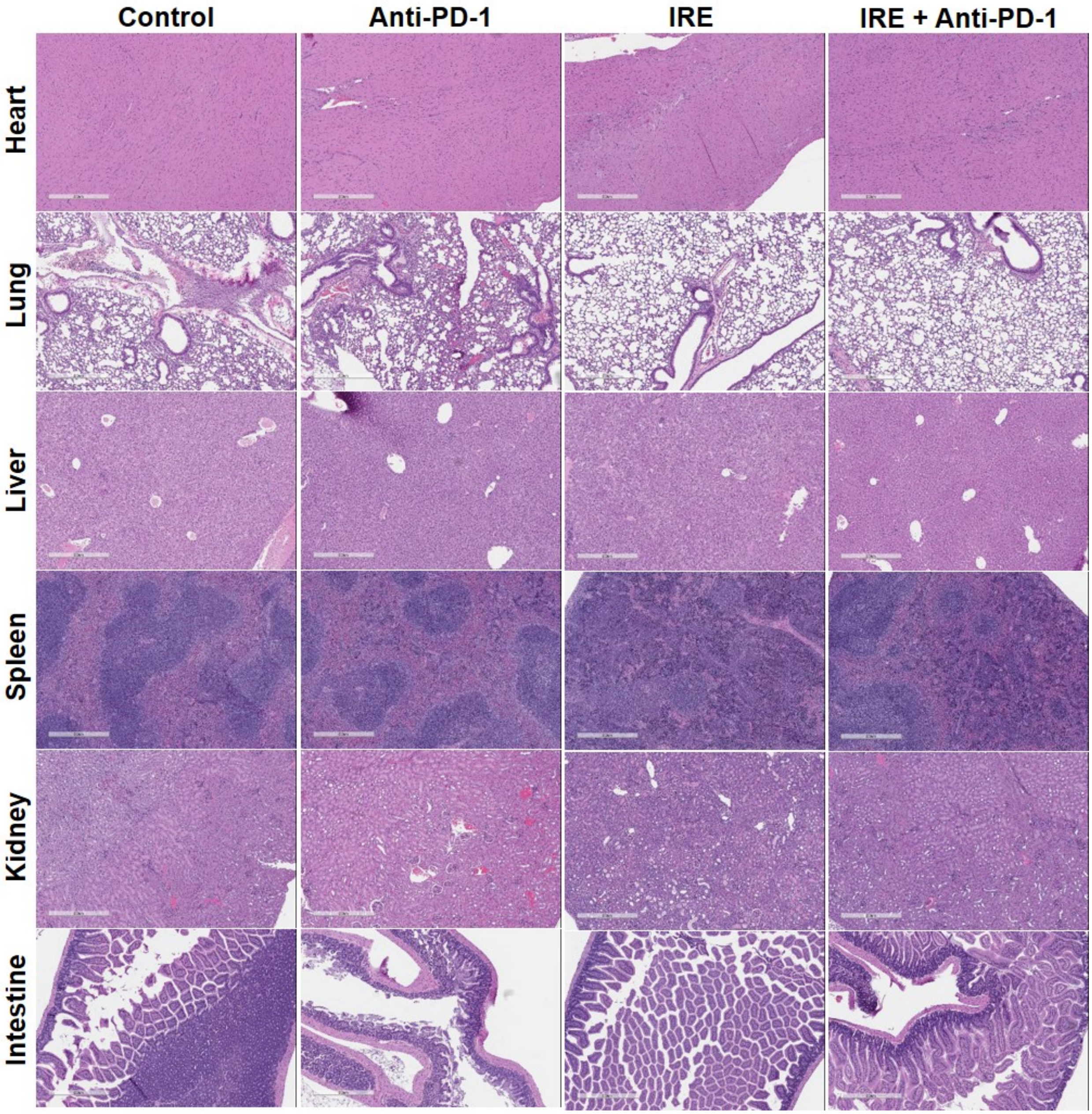

Supplementary Figure 2. H&E sections of non-tumor organs. Organs were removed and processed for H&E staining on day 9 after initiation of treatments. Scale bar = 2 mm.

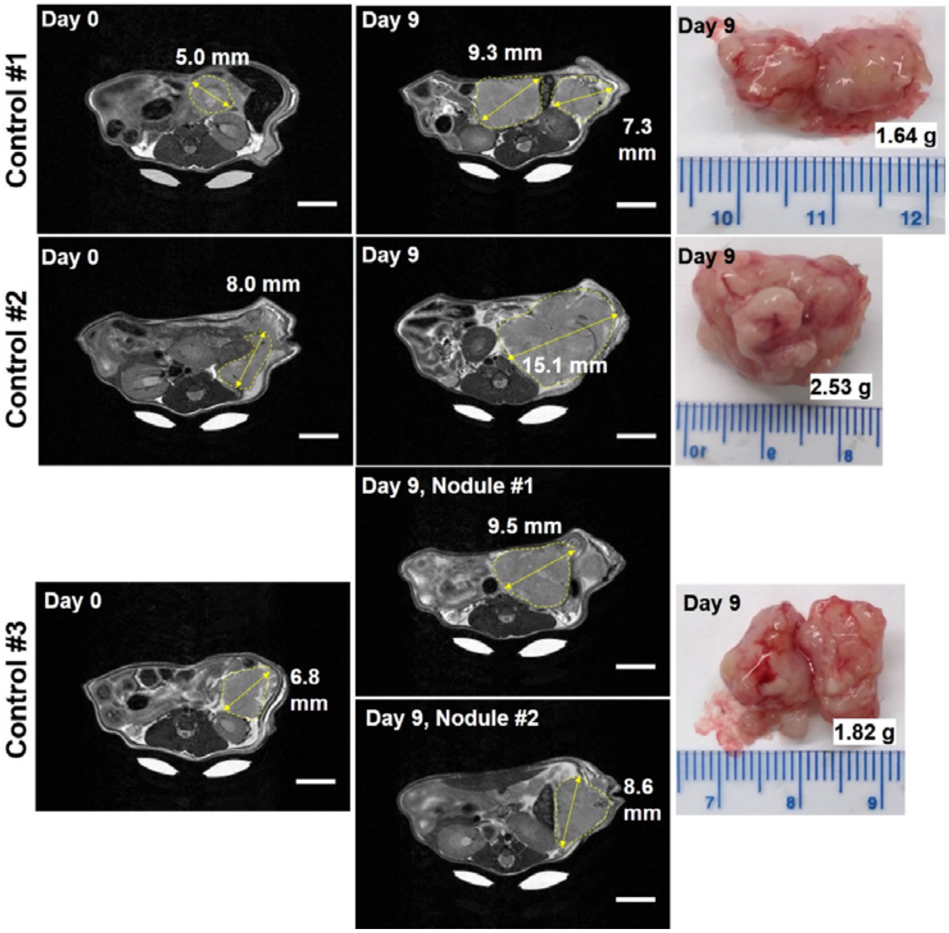

Supplementary Figure 3. T_2-MRI images of sham control KRAS* tumor and corresponding photographs. Three mice were imaged. Scale bar = 5 mm.

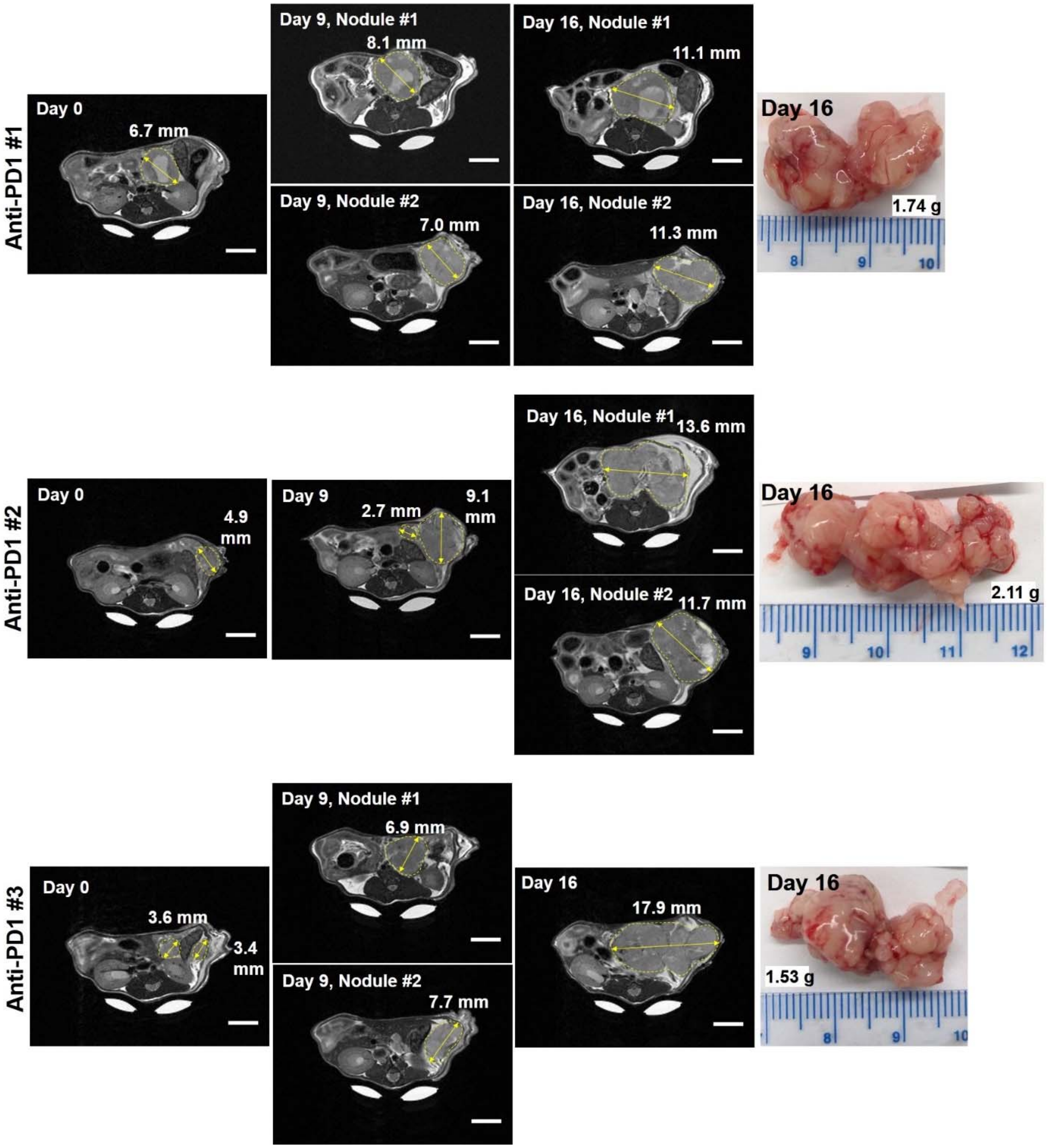

Supplementary Figure 4. T_2-MRI images of anti-PD1-treated KRAS* tumor and corresponding photographs. Anti-PD1 was intraperitoneally injected at 100 µg per mouse starting from day 0, then every 48 hours for 6 total doses. Three mice were imaged. Scale bar = 5 mm.

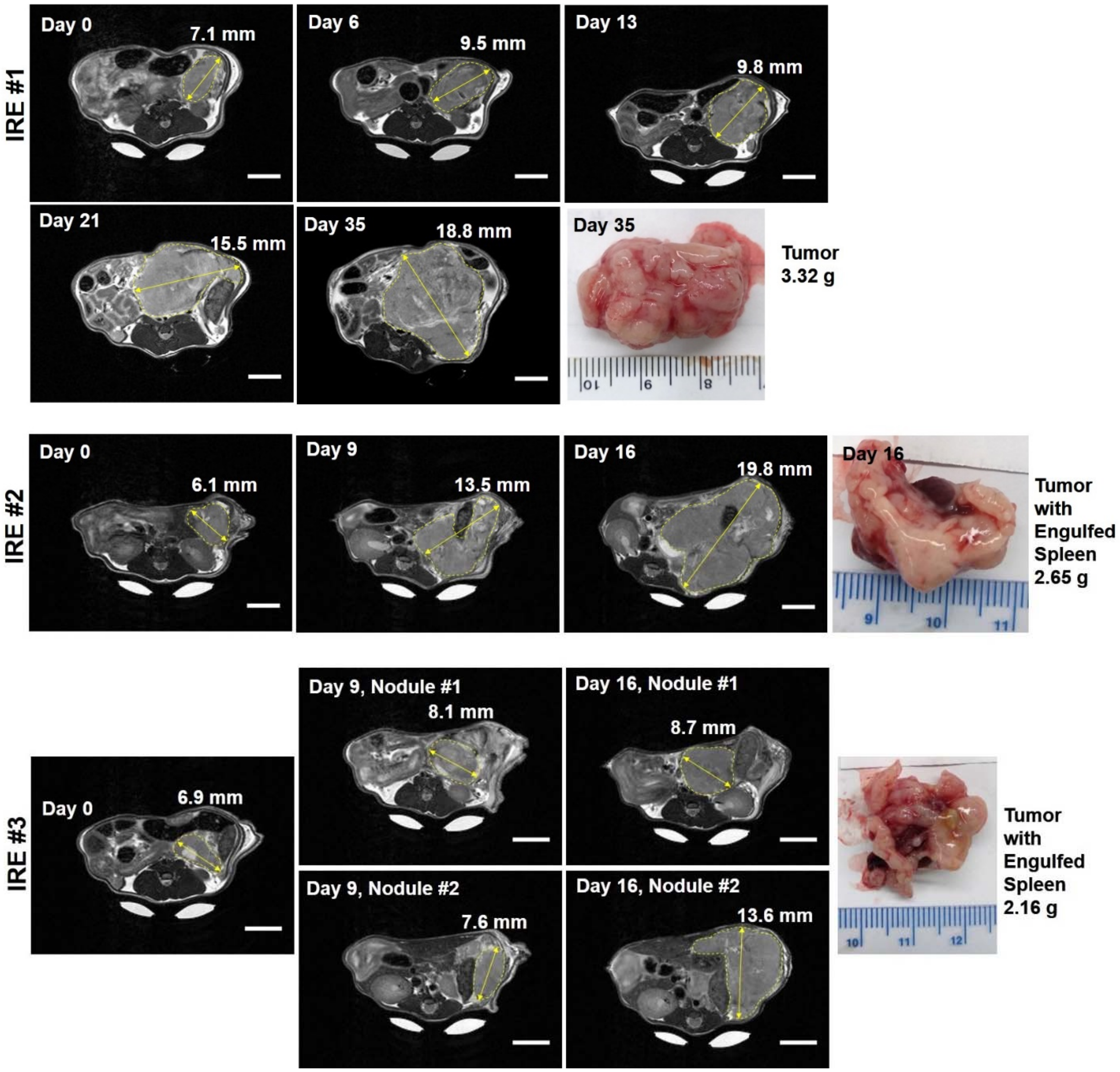

Supplementary Figure 5. T_2-MRI images of IRE-treated KRAS* tumor and corresponding photographs. IRE was conducted using a two-electrode array (5 mm between the electrodes). The array was inserted into the center of the tumor. The parameters of electric pulses were: 1200 V, 100 µsecond duration per pulse, 1 pulse per second, 99 pulses in total. Three mice were imaged. The day-0 images were acquired before institution of IRE. Scale bar = 5 mm.

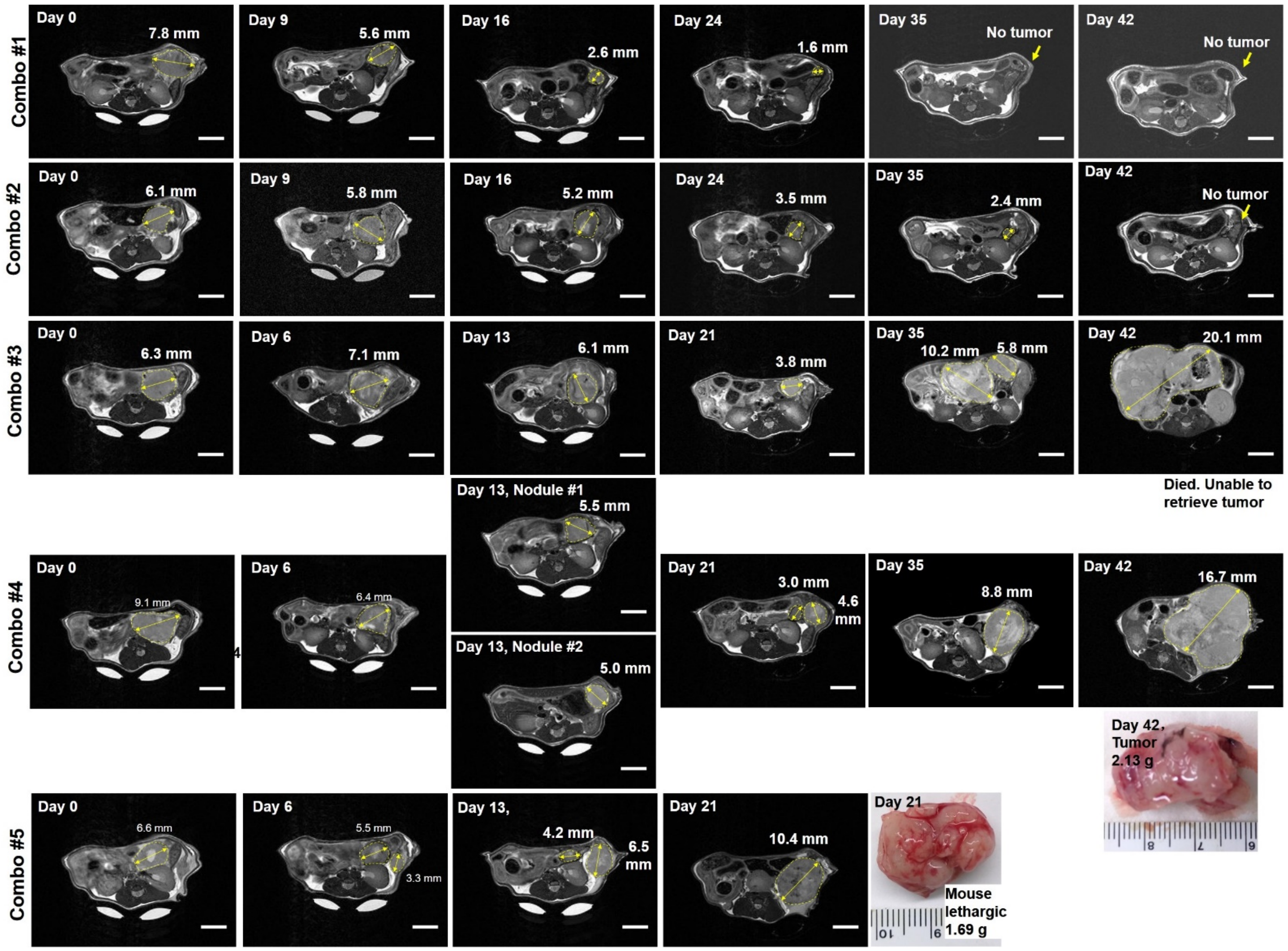

Supplementary Figure 6. T_2-MRI images of IRE + anti-PD1-treated KRAS* tumor and corresponding photographs. IRE was conducted using a two-electrode array with 5 mm between the electrodes. The array was inserted into the tumor center. The parameters of electric pulses were: 1200 V, 100 μsecond duration per pulse, 1 pulse per second, 99 pulses in total. Anti-PD1 was intraperitoneally injected at 100 μg per mouse starting from day 0, then every 48 hours for 6 total doses. The day-0 images were acquired before institution of IRE. Five mice were imaged. Scale bar = 5 mm.

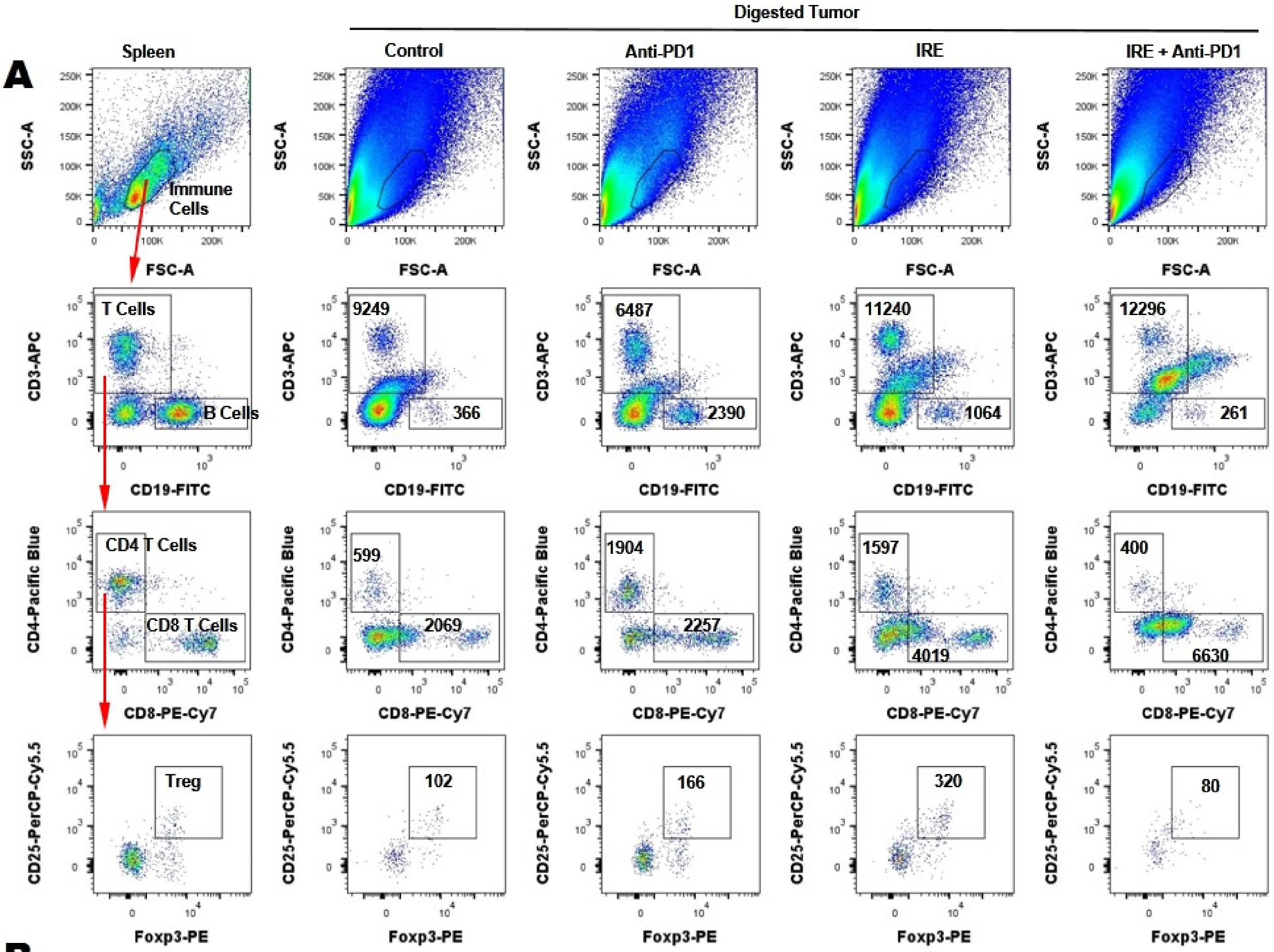

B

	A: Tumor Weight for Flow Cytometry (g)	B: Digested Tumor (μL)	C: Stained Tumor (μL)	D: Volume for Flow Cytometry (μL)	E: Actual Volume Analyzed (μL)	F: Normalization Factor = (D/E)x(B/C) /A	G: #CD8⁺ Cells	H: Normalized CD8⁺ Cell Frequency = FXG
Control	0.274	600	100	220	50	96.3	2068	$1.99x10^5$
Anti-PD1	0.264	600	100	220	50	100.0	2257	$2.26x10^5$
IRE	0.200	550	100	220	50	121.0	4019	$4.86x10^5$
IRE + Anti-PD1	0.069	600	100	220	50	382.6	6630	$2.54x10^6$

Supplementary Figure 7. Representative flow cytometry plots and calculations for Figs. 3b, d, e, f, and h. (**A**) Representative flow cytometry plots from 5 independent experiments per group. Splenocytes were analyzed in parallel to provide FSC/SSC gating for immune cells. Gating strategies are indicated with red arrows in the splenocytes column. The count of each cell populations are labeled in the corresponding box of the sham control, Anti-PD1, IRE, and IRE + Anti-PD1 groups. (**B**) Calculation formula. Use the $CD8^+$ T cells in the control group as an example. 0.274 gram of control tumor was digested and suspended in 600 μL staining buffer, out of which 100 μL was stained with antibodies. After washing, the cells were added to a well of 96-well U-bottom plate to a total volume of 220 μL. Then 50 μL cell suspension from each well was analyzed using flow cytometer.

A

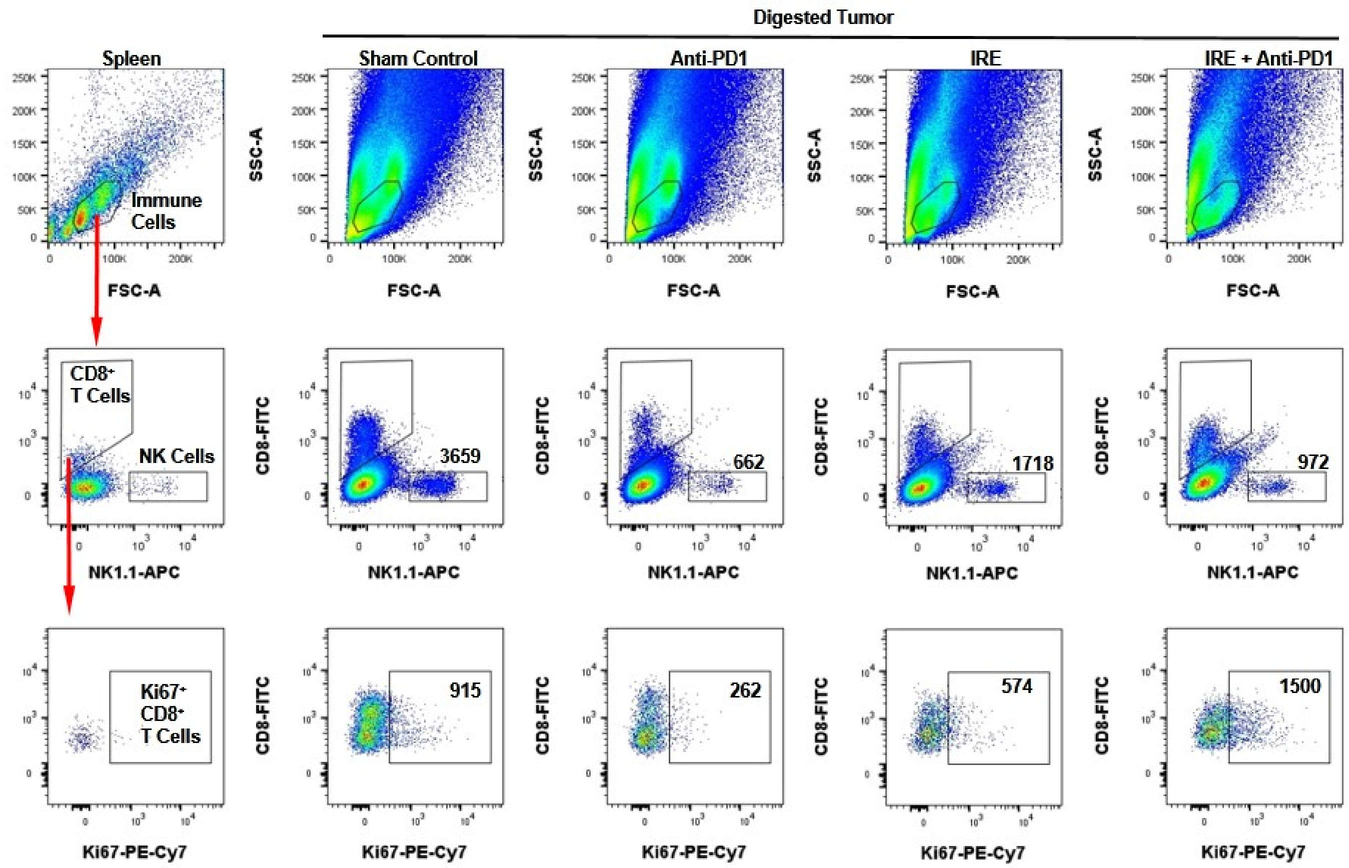

B

	A: Tumor Weight for Flow Cytometry (g)	B: Digested Tumor (µL)	C: Stained Tumor (µL)	D: Volume for Flow Cytometry (µL)	E: Actual Volume Analyzed (µL)	F: Normalization Factor = (D/E)x(B/C) /A	G: #CD8$^+$ Ki67$^+$ Cells	H: Normalized CD8$^+$Ki67$^+$Cell Frequency (#/g) = FXG
Control	0.418	600	100	220	50	63.1	915	5.78x10^4
Anti-PD1	0.358	600	100	220	50	73.7	262	1.93x10^4
IRE	0.271	600	100	220	50	97.4	574	5.59x10^4
IRE + Anti-PD1	0.107	600	100	220	50	246.7	1500	3.70x10^5

Supplementary Figure 8. Representative flow cytometry plots and calculations for Figs. 3c and g. (**A**) Representative flow cytometry plots from 5 independent experiments per group. Splenocytes were analyzed in parallel to provide FSC/SSC gating for immune cells. Gating strategies are indicated with red arrows in the splenocytes column. The counts of each cell populations are labeled in the corresponding box of the sham control, Anti-PD1, IRE, and IRE + Anti-PD1 groups. (**B**) Calculation formula. Use the CD8$^+$Ki67$^+$T cells in the control group as an example. 0.418 gram of control tumor was digested and suspended in 600 µL staining buffer, out of which 100 µL was stained with antibodies. After washing, the cells were added to a well of 96-well U-bottom plate to a total volume of 220 µL. Then 50 µL cell suspension from the well was analyzed using flow cytometer.

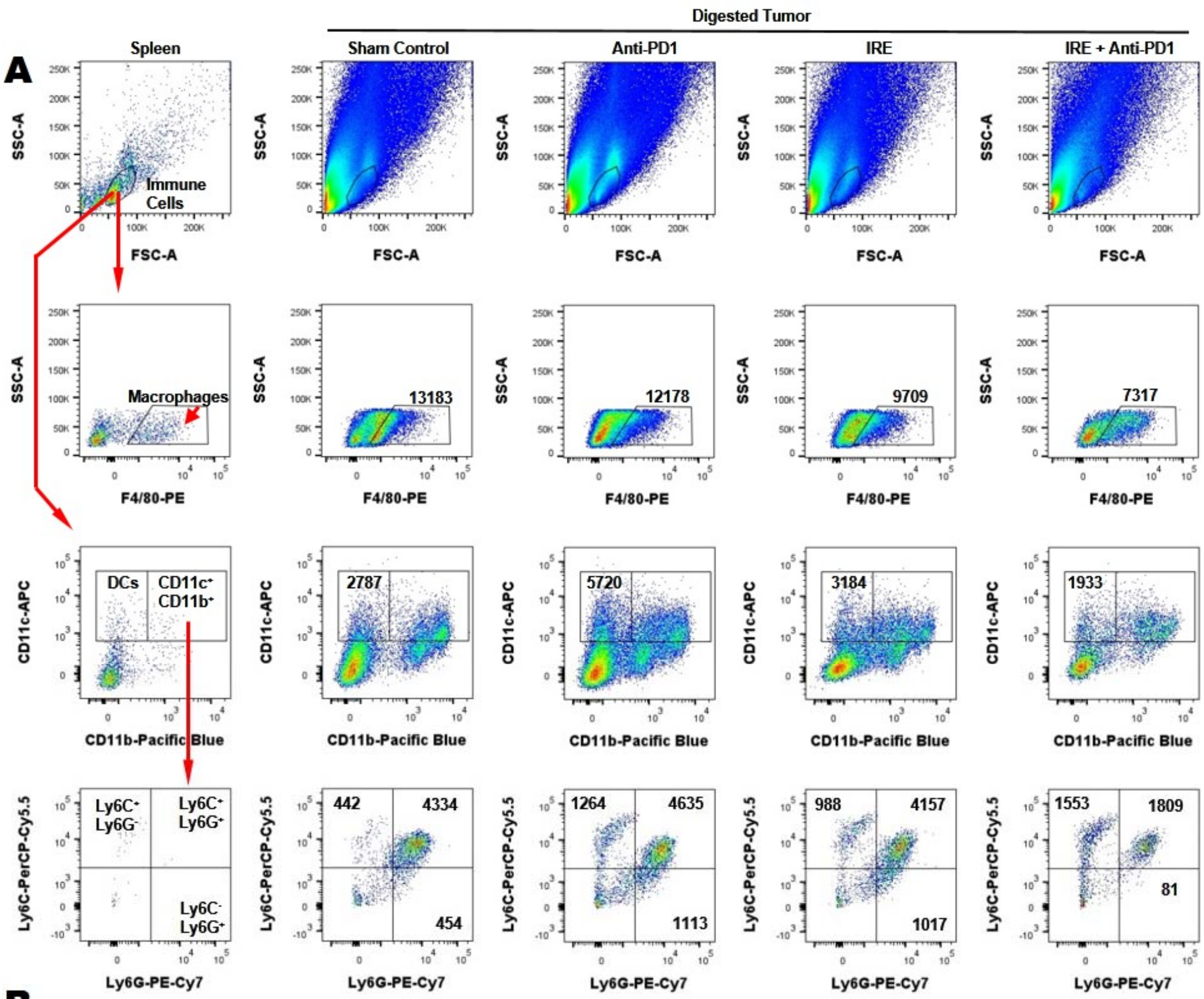

B

	A: Tumor Weight for Flow Cytometry (g)	B: Digested Tumor (µL)	C: Stained Tumor (µL)	D: Volume for Flow Cytometry (µL)	E: Actual Volume Analyzed (µL)	F: Normalization Factor = (D/E)x(B/C) /A	G: #DCs Cells	H: Normalized DC Cell Frequency (#/g) = FXG
Control	0.274	600	100	220	50	96.3	2787	2.69×10^5
Anti-PD1	0.264	600	100	220	50	100.0	5720	5.72×10^5
IRE	0.200	550	100	220	50	121.0	3184	3.85×10^5
IRE + Anti-PD1	0.083	600	100	220	50	318.1	1933	6.15×10^5

Supplementary Figure 9. Representative flow cytometry plots and calculations for Figs. 3i, j, and k. (**A**) Representative flow cytometry plots from 5 independent experiments per group. Splenocytes were analyzed in parallel to provide FSC/SSC gating for immune cells. Gating strategies are indicated with red arrows in the splenocytes column. The count of each cell populations are labeled in the corresponding box of the sham control, Anti-PD1, IRE, and IRE + Anti-PD1 groups. (**B**) Calculation formula. Use the $CD11c^+CD11b^-$ dendritic cells (DCs) in the control group as an example. 0.274 gram of control tumor was digested and suspended in 600 µL staining buffer, out of which 100 µL was stained with antibodies. After washing, the cells were added to a well of 96-well U-bottom plate to a total volume of 220 µL. Then 50 µL cell suspension from the well was analyzed using flow cytometer.

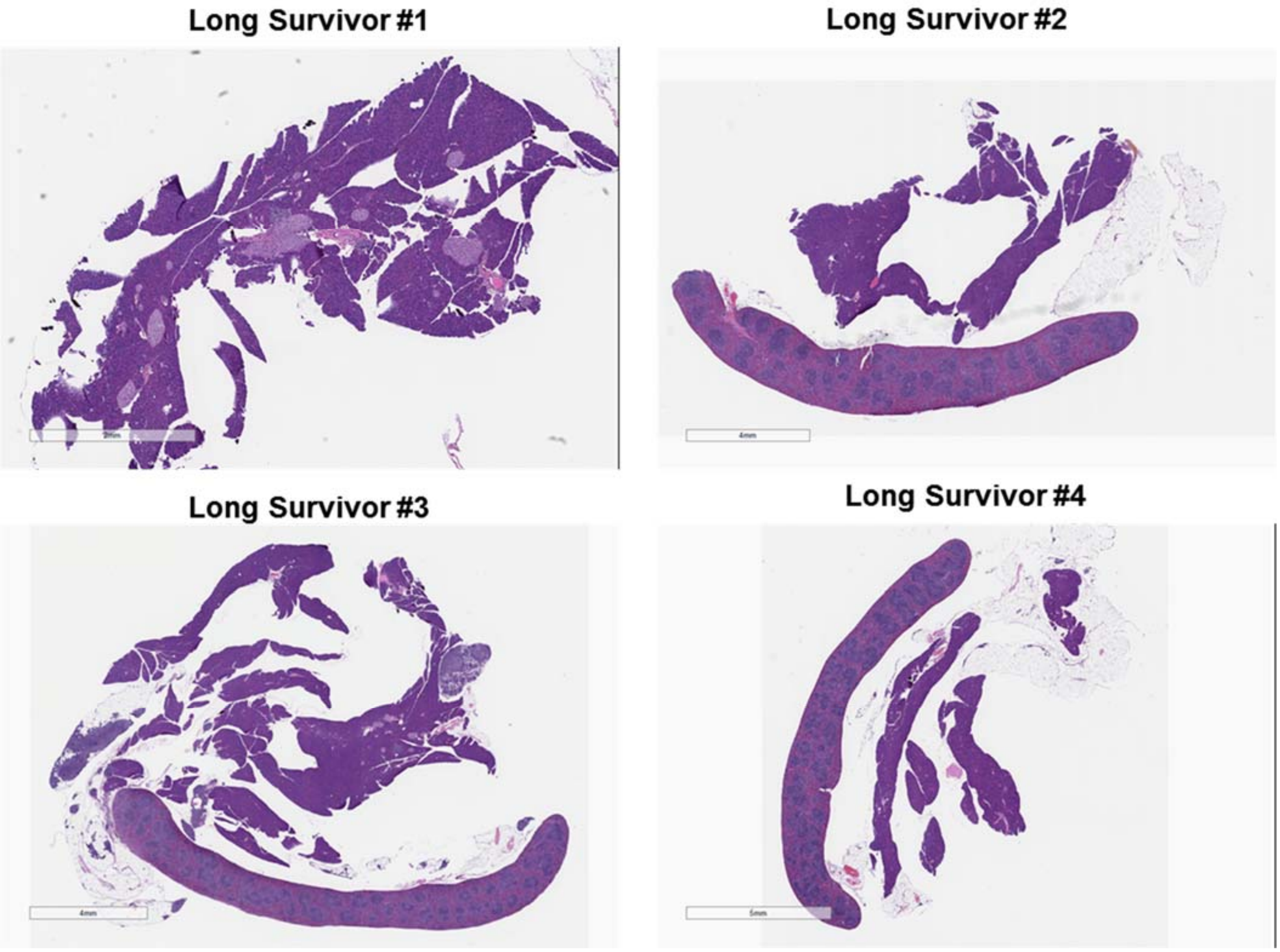

Supplementary Figure 10. Overview of hematoxylin-eosin stained pancreas and/or spleen of KRAS*-bearing mice that received IRE + anti-PD1 treatment, and had survived for more than 9 months.

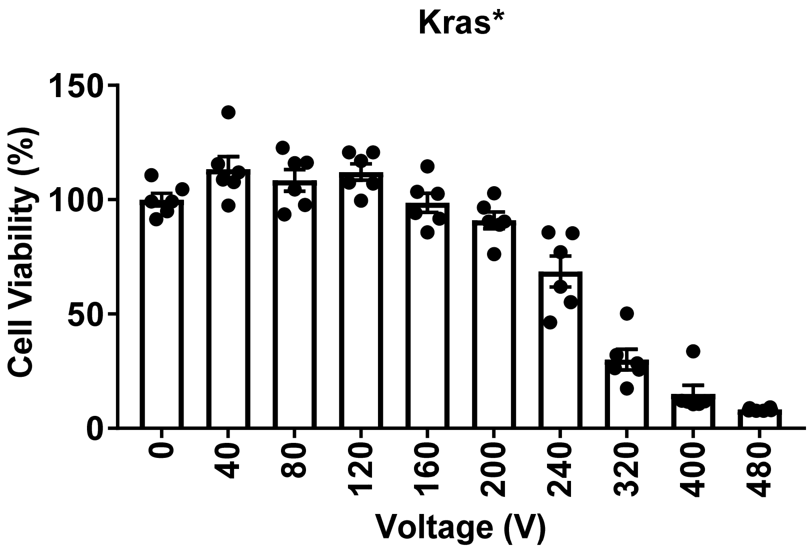

Supplementary Figure 11. KRAS* cell viability after treatment with electric pulses at different voltages. Kras* cells at 1 million cells/mL in PBS were added to 4-mm gap cuvette and subjected to electroporation from 0 to 480 V pulses with the following parameter: duration 100 μsecond, repetition frequency 1 Hz, number of pulses 20. Cells suspension was diluted with DMEM/F-12 (0.5%FBS) to 10,000 cells/mL. 100 μL was added to a well of 96-well plate, and cultured for 48 hours. Cell viability was measured with crystal violet staining and normalized to control with 0 V sham experiment. Data are presented as mean ± standard deviation of mean (N = 6). Source data are provided as a Source Data file.

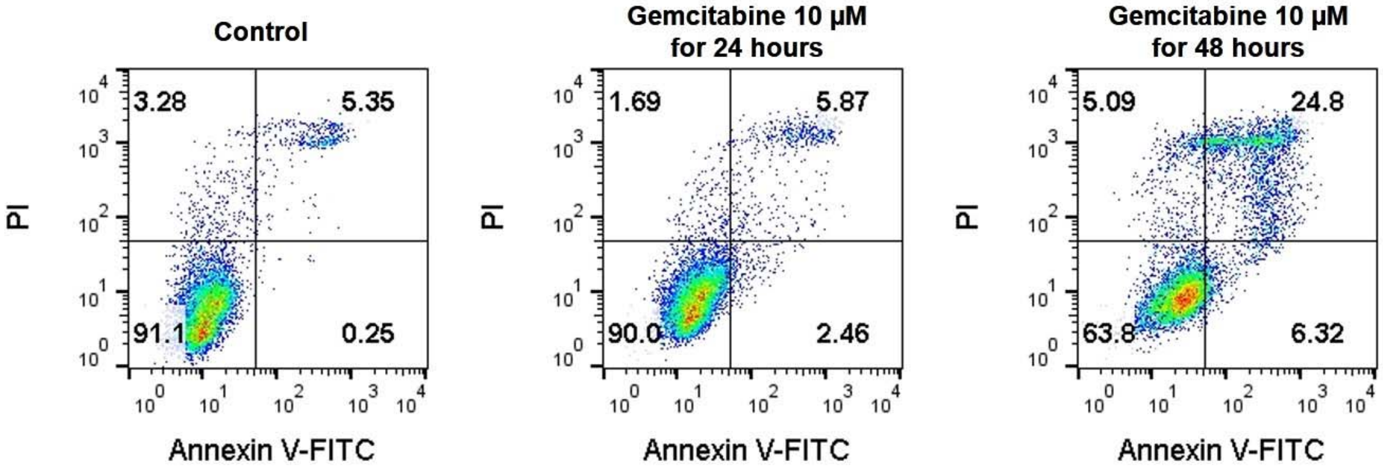

Supplementary Figure 12. Representative flow cytometry plot of gemcitabine-treated KRAS* cells. Cells were treated with 10 μM Gemcitabine at 37 °C. Significant cell apoptosis and necrosis occurred after 48 hours of treatment. Three independent experiments were performed.

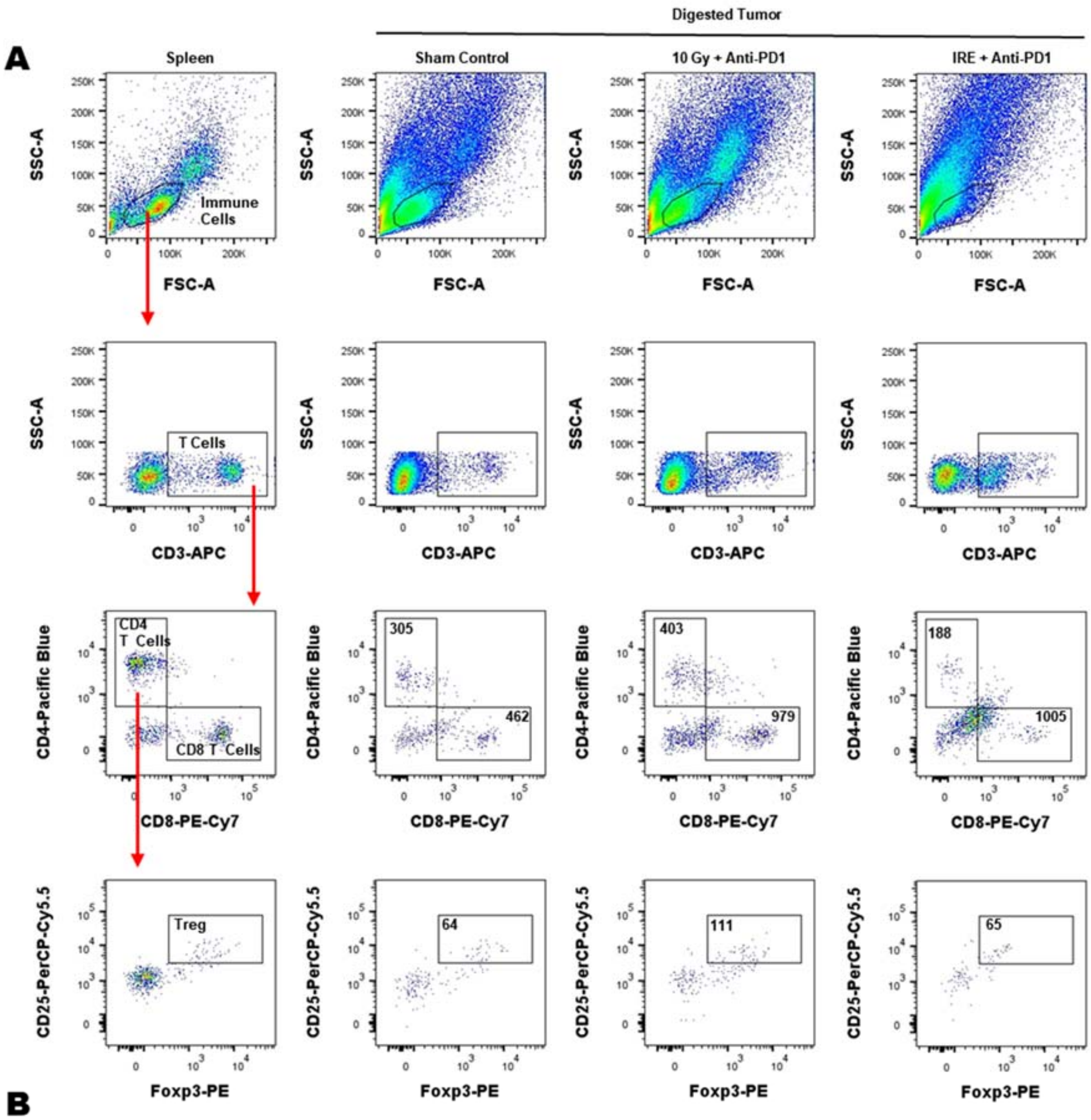

B

	A: Tumor Weight for Flow Cytometry (g)	B: Digested Tumor (μL)	C: Stained Tumor (μL)	D: Volume for Flow Cytometry (μL)	E: Additional Dilution ratio	F: Actual Volume Analyzed (μL)	G: Normalization Factor = (DxE/F)x(B/C) /A	H: #CD8⁺ Cells	I: Normalized DC Cell Frequency (#/g) = GXH
Control	0.299	550	100	200	10	50	735.8	462	3.40×10^5
10 Gy + Anti-PD1	0.292	550	100	200	10	50	753.4	979	7.38×10^5
IRE + Anti-PD1	0.126	550	100	200	10	50	1990.5	1005	2.00×10^6

Supplementary Figure 13. Representative flow cytometry plots and calculations for Figs. 8g, h, and i. (**A**) Representative flow cytometry plots from 4 to 5 independent experiments per group. Splenocytes were analyzed in parallel to provide FSC/SSC gating for immune cells. Gating strategies are indicated with red arrows in the splenocytes column. The count of each cell populations are labeled in the corresponding box of the sham control, Anti-PD1, IRE, and IRE + Anti-PD1 groups. (**B**) Calculation formula. Use the $CD8^+$ T cells in the control group as an example. 0.299 gram of control tumor was digested and suspended in 550 μL staining buffer, out of which 100 μL was stained with antibodies. After washing, the cells were added to a well of 96-well U-bottom plate to a total volume of 200 μL. Additional 1-to-10 dilution was performed due to sensor saturation during initial run. Then 50 μL of diluted cell suspension from the well was analyzed using flow cytometer.

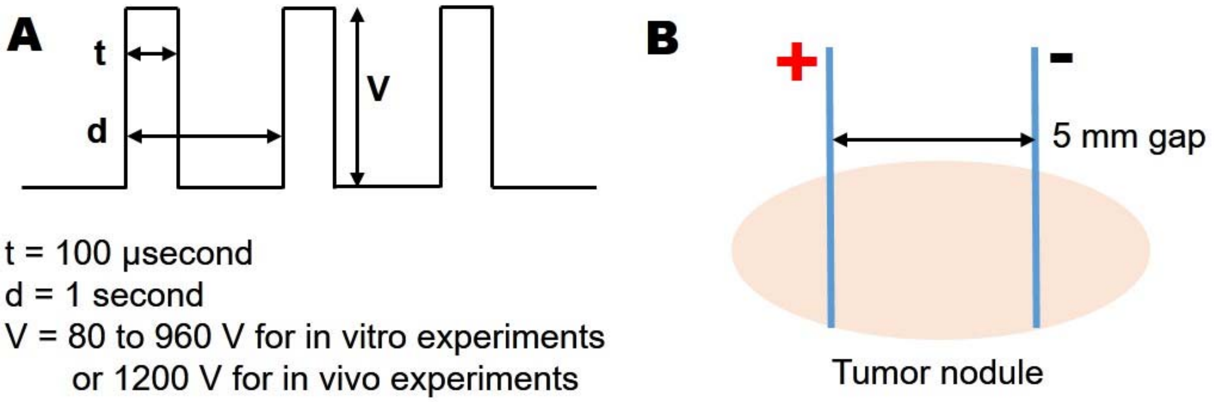

Supplementary Figure 14. Experiment set up for electroporation. (**A**) Parameters for the electric pulses. (**B**) Side view of the placement of the two-needle electrode for in vivo experiment.

(A) HIF-1α, Novus Biological, NB100-134, 1:500

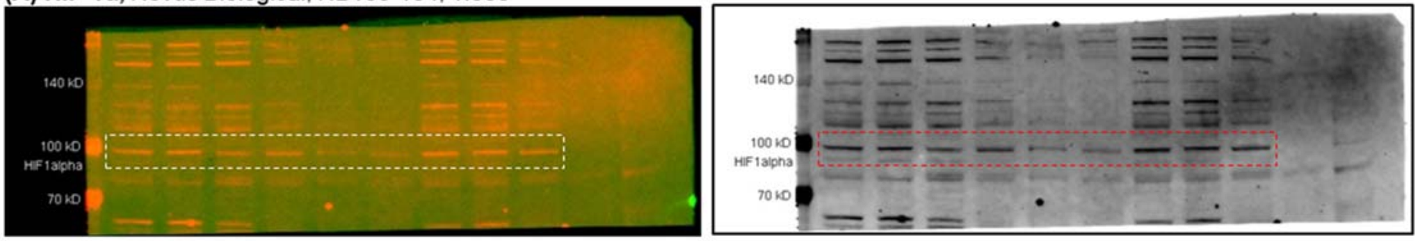

(B) CA-IX, Santa Cruz, sc-17253, 1:500

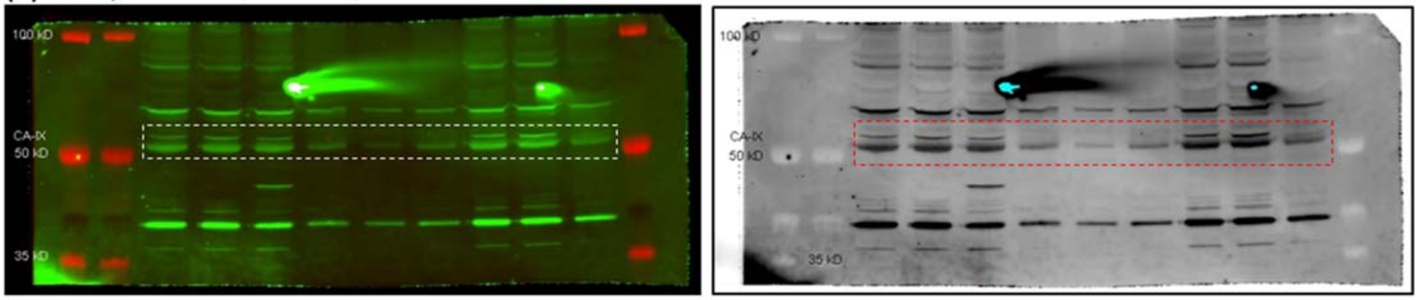

(C) Alpha-SMA, Abcam, ab5694, 1:1000

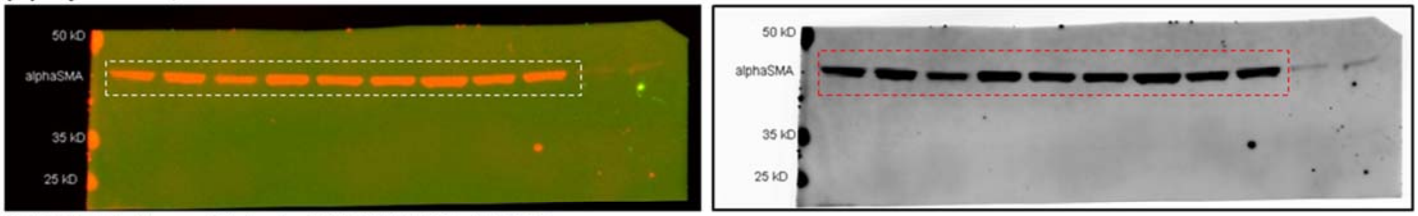

(D) HABP1, Novus Biological, NBP1-89790, 1:1000

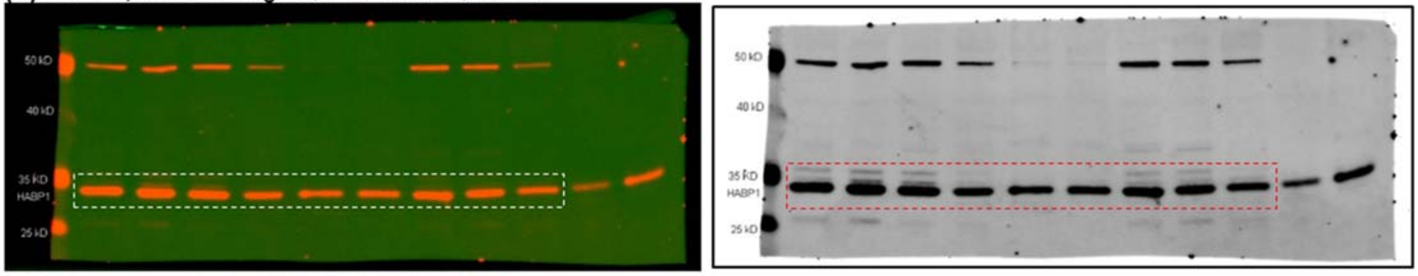

(E) LOX, Novus Biological, NB100-2527, 1:500

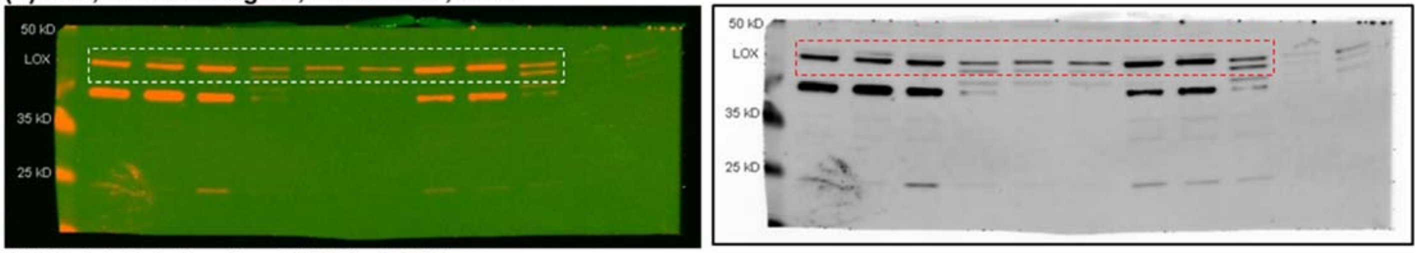

(F) PD-L1, Cell Signaling, #13684, 1:1000

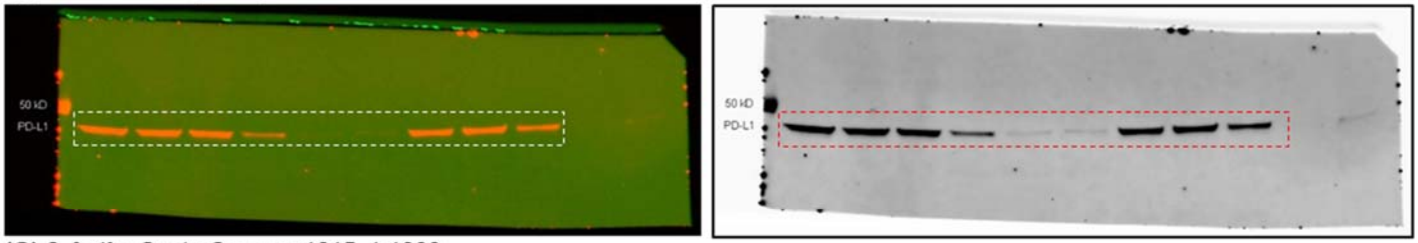

(G) β-Actin, Santa Cruz, sc-1615, 1:1000

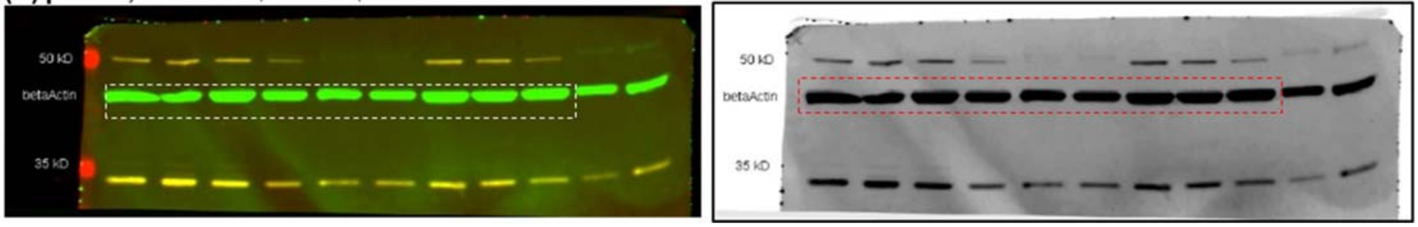

Supplementary Figure 15. Original un-cut western blot images for Fig. 6d. Bands of interest are circled with dashed lines. Western images were acquired in 700-nm (red) and 800-nm (green) channels on an Odyssey near-infrared fluorescence scanner (LI-COR). The original dual-color images are displayed in the left column. The target channel signals were then converted to gray scale and shown as monochrome images in the right column. Molecular weight markers are labeled along with the target proteins.

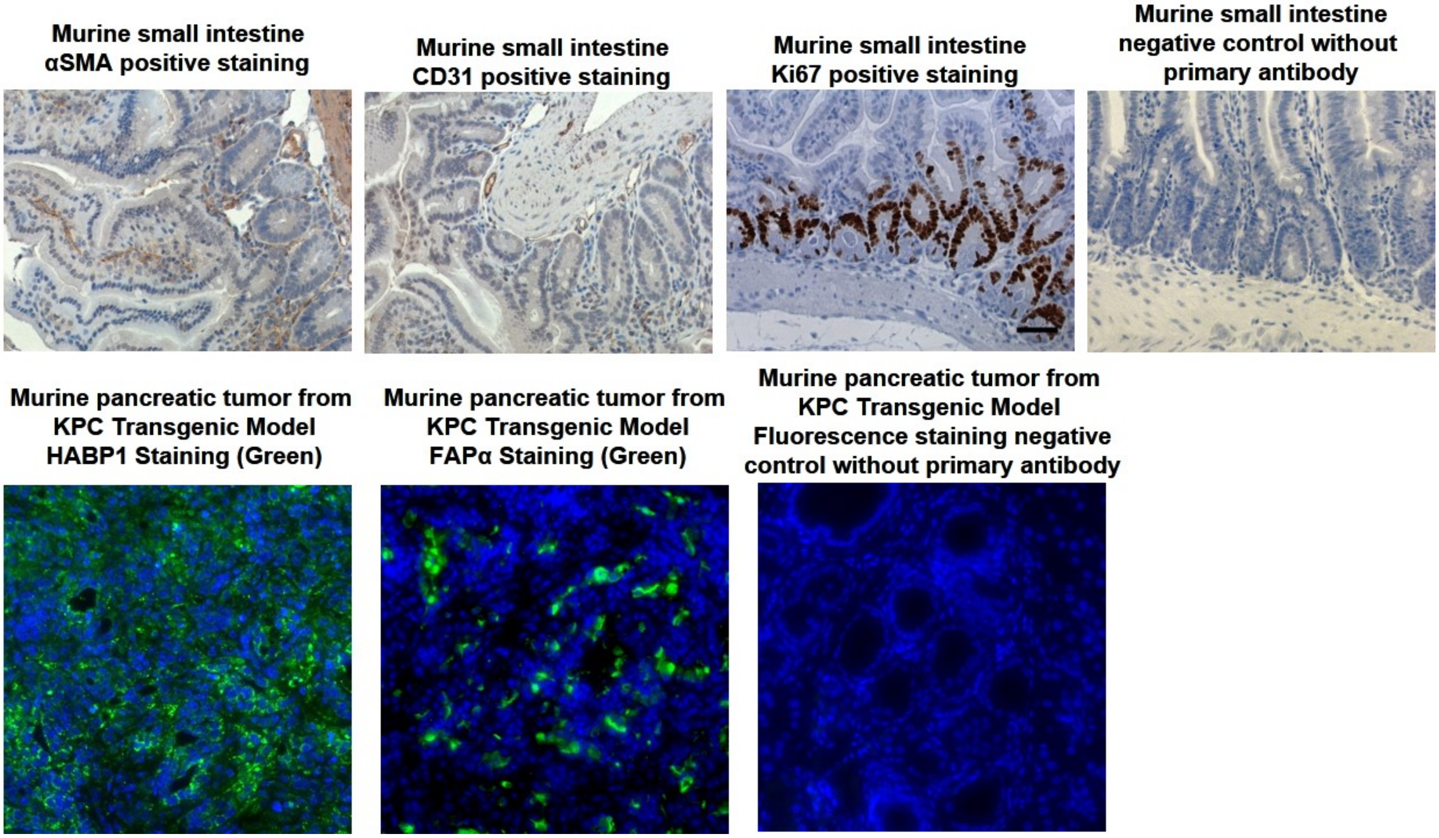

Supplementary Figure 16. Positive and negative controls for IHC staining.

Supplementary Table 1. Primary antibody information for WB and IHC

Target	Host	Source and Catalog Number	Application
Carbonic anhydrases IX (N-19)	Goat	Santa Cruz, sc-17253	WB (1:500 dilution)
CD31	Rabbit	Abcam, 28364	IHC (1:50 dilution)
Fibroblast activation protein alpha	Rabbit	Abcam, ab28244	IHC (1:100 dilution)
HIF-1 alpha	Rabbit	Novus Biological, NB100-134	WB (1:1000 dilution)
PD-L1	Rabbit	Cell Signaling, 13684	WB (1:100 dilution)
Hyaluronan-binding protein 1 (HABP1)	Rabbit	Novus Biological, NBP1-89790	IHC (1:300 dilution),<br>WB (1:1000 dilution)
Ki67 (mouse specific)	Rabbit	Cell Signaling, #12202	IHC (1:400 dilution)
Lysyl oxidase (LOX)	Rabbit	Novus Biological, NB100-2527	WB (1:300 dilution)
α-Smooth muscle actin	Rabbit	Abcam, ab5694	IHC (1:200 dilution),<br>WB (1:1000 dilution)
β-Actin	Goat	Santa Cruz, sc-1615	WB (1:1000 dilution)

IHC: Immunohistochemical staining. WB: western blotting.
