# Supplementary material for: Irreversible electroporation reverses resistance to immune checkpoint blockade in pancreatic cancer
Source: Nat Commun. 2019 Feb 22;10:899. doi: 10.1038/s41467-019-08782-1 (PMC6385305; doi:10.1038/s41467-019-08782-1)
Supplement: Supplementary file 2 — Reporting Summary [file 41467_2019_8782_MOESM2_ESM.pdf]

nature research

Corresponding author(s): Chun Li

Last updated by author(s): Jan 10, 2019

Reporting Summary

Nature Research wishes to improve the reproducibility of the work that we publish. This form provides structure for consistency and transparency in reporting. For further information on Nature Research policies, see Authors & Referees and the Editorial Policy Checklist.

Statistics

For all statistical analyses, confirm that the following items are present in the figure legend, table legend, main text, or Methods section.

n/a	Confirmed	
☐	☒	The exact sample size (*n*) for each experimental group/condition, given as a discrete number and unit of measurement
☐	☒	A statement on whether measurements were taken from distinct samples or whether the same sample was measured repeatedly
☐	☒	The statistical test(s) used AND whether they are one- or two-sided<br>*Only common tests should be described solely by name; describe more complex techniques in the Methods section.*
☐	☒	A description of all covariates tested
☐	☒	A description of any assumptions or corrections, such as tests of normality and adjustment for multiple comparisons
☐	☒	A full description of the statistical parameters including central tendency (e.g. means) or other basic estimates (e.g. regression coefficient) AND variation (e.g. standard deviation) or associated estimates of uncertainty (e.g. confidence intervals)
☐	☒	For null hypothesis testing, the test statistic (e.g. *F*, *t*, *r*) with confidence intervals, effect sizes, degrees of freedom and *P* value noted<br>*Give P values as exact values whenever suitable.*
☒	☐	For Bayesian analysis, information on the choice of priors and Markov chain Monte Carlo settings
☒	☐	For hierarchical and complex designs, identification of the appropriate level for tests and full reporting of outcomes
☒	☐	Estimates of effect sizes (e.g. Cohen's *d*, Pearson's *r*), indicating how they were calculated

Our web collection on statistics for biologists contains articles on many of the points above.

Software and code

Policy information about availability of computer code

Data collection	Flow cytometry data was collected using DIVA 5.0 software. Microscopy images were taken using AxioVision Microscope Software 4.8
Data analysis	Flow cytometry data was analyzed using Flow Jo V10. Microscopy images were analyzed using AxioVision Microscope Software 4.8 and ImageJ 1.52a

For manuscripts utilizing custom algorithms or software that are central to the research but not yet described in published literature, software must be made available to editors/reviewers. We strongly encourage code deposition in a community repository (e.g. GitHub). See the Nature Research guidelines for submitting code & software for further information.

Data

Policy information about availability of data

All manuscripts must include a data availability statement. This statement should provide the following information, where applicable:

- Accession codes, unique identifiers, or web links for publicly available datasets
- A list of figures that have associated raw data
- A description of any restrictions on data availability

All the data supporting the findings of this study are presented within the article and/or its Supplementary Information. The source data underlying Figures 1-9, and Supplementary Figures 1 and 11, are provided with the paper as a Source Data file.

Field-specific reporting

Please select the one below that is the best fit for your research. If you are not sure, read the appropriate sections before making your selection.

☒ Life sciences ☐ Behavioural & social sciences ☐ Ecological, evolutionary & environmental sciences

For a reference copy of the document with all sections, see nature.com/documents/nr-reporting-summary-flat.pdf

Life sciences study design

All studies must disclose on these points even when the disclosure is negative.

Sample size	The sample size in each experiment was determined to give an appropriate power of the test
Data exclusions	No data was excluded
Replication	All attempts at replication were successful as determined using a statistical analysis
Randomization	Allocation was random in all of the experiments
Blinding	All experiments were conducted in a double blinded fashion in which the researchers were blinded to group allocation.

Reporting for specific materials, systems and methods

We require information from authors about some types of materials, experimental systems and methods used in many studies. Here, indicate whether each material, system or method listed is relevant to your study. If you are not sure if a list item applies to your research, read the appropriate section before selecting a response.

Materials & experimental systems

n/a	Involved in the study	
☐	☒	Antibodies
☐	☒	Eukaryotic cell lines
☒	☐	Palaeontology
☐	☒	Animals and other organisms
☒	☐	Human research participants
☒	☐	Clinical data

Methods

n/a	Involved in the study	
☒	☐	ChIP-seq
☐	☒	Flow cytometry
☒	☐	MRI-based neuroimaging

Antibodies

Antibodies used	Flow cytometry antibodies: CD11b-Pacific Blue (clone M1/79, Cat#101223), Ly6C-PerCP-Cy5.5 (clone AL-21, Cat#560525), Ly6G-PE-Cy7 (clone 1A8, Cat#127617), CD19-FITC (clone 1D3, Cat#152403), CD8α-PE-Cy7 (clone 53-6.7, Cat#100721), F4/80-PE or F4/80-APC-Cy7 (clone BM8, Cat#123109, Cat#123117), CD4-Pacific Blue or CD4-PerCP-Cy5.5 (clone GK1.5, Cat#100427, Cat#100431), NK1.1-APC (clone PK136, Cat#108709), CD11c-APC or CD11c PerCP-Cy5.5 (clone N418, Cat#117309, Cat#117327), CD62L-PE (clone MEL-14 Cat#104407), CD44-FITC (clone IM7, Cat#103021), CD86-PE-Cy7 (clone GL-1, Cat#105013), CD40-APC (clone 3/23, Cat#124611), CCR7-PE (clone 4B12, Cat#120105), MHC-II-FITC (clone M5/114.15.2, Cat#107605), and Ki67-PE-Cy7 (Clone 16AB, Cat#652425) were obtained from BioLegend (San Jose, CA). Foxp3-PE (clone FJK-16s, #12-5773-82) was obtained from eBioscience (Thermo Fisher Scientific, Waltham, MA).<br><br>IHC and western blot antibodies: CA-IX (Santa Cruz, sc-17253), CD31 (Abcam 28364), FAPalpha (Abcam 28244), HIF-1alpha (Novus Biological, NB100-134), HABP1 (Novus Biological, NBP1-89790), Ki67 (mouse specific) (Cell Signaling, #12202), alphaSMA (Abcam, ab5694).
Validation	Validation of flow cytometry antibodies are provided at manufacturer's website. Positive and negative controls were used for IHC staining antibodies, see Supplementary Data.

Eukaryotic cell lines

Policy information about cell lines

Cell line source(s)	B16F10 was purchased from ATCC. KRAS* was provided by Dr. Y Alan Wang at the University of Texas MD Anderson Cancer Center
Authentication	The cell lines used here were validated by short tandem repeat (STR) DNA fingerprinting by the MD Anderson Cancer Center

Authentication	Characterized Cell Line Core using the AmpFLSTR identifier kit according to manufacturer's instructions (Applied Biosystems, Thermo Scientific, Rockford, IL). The STR profiles were compared to known ATCC fingerprints (ATCC.org), to the Cell Line
Mycoplasma contamination	All cell lines were routinely checked every two months for Mycoplasma contamination
Commonly misidentified lines (See ICLAC register)	No misidentified lines were used.

Animals and other organisms

Policy information about studies involving animals; ARRIVE guidelines recommended for reporting animal research

Laboratory animals	C57BL/6 mice from Taconic Bioscience (#B6-F), 8 weeks of age, Female
Wild animals	No wild animals were used
Field-collected samples	No filed-collection was performed
Ethics oversight	All animal studies comply with relevant ethical regulations for animal testing and research, and were approved by the Institutional Animal Care and Use Committee of The University of Texas MD Anderson Cancer Center. Animals were maintained and studies were carried out in accordance with institutional guidelines.

Note that full information on the approval of the study protocol must also be provided in the manuscript.

Flow Cytometry

Plots

Confirm that:

☒ The axis labels state the marker and fluorochrome used (e.g. CD4-FITC).

☒ The axis scales are clearly visible. Include numbers along axes only for bottom left plot of group (a 'group' is an analysis of identical markers).

☒ All plots are contour plots with outliers or pseudocolor plots.

☒ A numerical value for number of cells or percentage (with statistics) is provided.

Methodology

Sample preparation	(1) Cultured cells were trypsinized and stained with Annexin V-FITC/PI to determine the percentages of apoptosis and necrosis.<br>(2) For analyses of intratumoral immune cells, weighed tumors were minced and digested in an 8-mL mixture of 2 mg/mL collagenase type IV (LS004188; Worthington, Lakewood, NJ), 0.2 mg/mL hyaluronidase (H3506, Sigma-Aldrich, St Louis, MO), and 0.2 mg/mL DNase I (D4527, Sigma-Aldrich) in DMEM/F12 medium at 37°C for 30 min. The mixture was shaken constantly at 20 RPM. Debris was removed by filtration through a 40-µm mesh, and the red blood cells were removed with a red blood cell lysis buffer (R7757, Sigma-Aldrich). The mixture was pelleted and re-suspended in PBS supplemented with 2% fetal bovine serum for further analyses.<br>(3) For splenocytes, spleens were minced and passed through a 40-µm mesh, and the red blood cells were removed with a red blood cell lysis buffer (R7757, Sigma-Aldrich). The mixture was pelleted and re-suspended in PBS supplemented with 2% fetal bovine serum for further analyses.
Instrument	LSRFortessa X-20
Software	Data was collected using BD FACSDIVA software, and analyzed using Flow Jo V10
Cell population abundance	No sorting was involved
Gating strategy	Intratumoral immune cells were gated using a parallel splenocyte sample on FSC/SCC. Specific cell populations were then identified using corresponding antibodies: CD8+ T cells (CD3+CD8+); proliferating CD8+ T cells (CD8+Ki67+); CD4+ T cells (CD3 +CD4+); Tregs (CD3+CD4+CD25+Foxp3+); NK cells (NK1.1+); B cells (CD19+); DCs (CD11c+CD11b-); MDSCs (CD11c+CD11b+Ly6C+ or CD11c+CD11b+Ly6G+).

☒ Tick this box to confirm that a figure exemplifying the gating strategy is provided in the Supplementary Information.
